# Genetic, metabolic and clinical delineation of an *MRPS23*-associated mitochondrial disorder

**DOI:** 10.1038/s41598-023-49161-7

**Published:** 2023-12-12

**Authors:** Chupong Ittiwut, Rungnapa Ittiwut, Chulaluck Kuptanon, Tetsuro Matsuhashi, Masaru Shimura, Yohei Sugiyama, Takanori Onuki, Akira Ohtake, Kei Murayama, Nithiwat Vatanavicharn, Waralee Dejputtawat, Nitchanund Tantisirivit, Phawin Kor-anantakul, Wuttichart Kamolvisit, Kanya Suphapeetiporn, Vorasuk Shotelersuk

**Affiliations:** 1 Center of Excellence for Medical Genomics, Department of Pediatrics, Faculty of Medicine, Chulalongkorn University, Bangkok, Thailand; 2 Excellence Center for Genomics and Precision Medicine, King Chulalongkorn Memorial Hospital, The Thai Red Cross Society, Bangkok, Thailand; 3 Department of Pediatrics, Queen Sirikit National Institute of Child Health, Bangkok, Thailand; 4 Center for Medical Genetics and Department of Metabolism, Chiba Children’s Hospital, Chiba, Japan; 5 Center for Intractable Diseases, Saitama Medical University Hospital, Saitama, Japan; 6 Division of Medical Genetics, Department of Pediatrics, Siriraj Hospital, Mahidol University, Bangkok, Thailand; 7 Division of Growth and Development, Department of Pediatrics, Nakornping Hospital, Chiang Mai, Thailand; 8 Department of Pediatrics, Nan Hospital, Nan, Thailand

**Keywords:** Genetics, Medical research, Molecular medicine

## Abstract

*MRPS23* is a nuclear gene encoding a mitochondrial ribosomal protein. A patient with a mitochondrial disorder was found to carry a variant in *MRPS23*. More cases are necessary to establish *MRPS23* as a mitochondrial disease gene. Of 5134 exomes performed in our center, we identified five independent patients who had similar clinical manifestations and were homozygous for the same germline variant c.119C>T; p.P40L in *MRPS23*. Detailed clinical findings, mitochondrial enzyme activity assays from cultured skin fibroblasts, PCR-Sanger-sequencing, and variant age estimation were performed. Their available family members were also studied. Eight members homozygous for the *MRPS23* p.P40L were identified. All were from Hmong hilltribe. Seven presented with alteration of consciousness and recurrent vomiting, while the eighth who was a younger brother of a proband was found pre-symptomatically. Patients showed delayed growth and development, hearing impairment, hypoglycemia, lactic acidosis, and liver dysfunction. In vitro assays of cultured fibroblasts showed combined respiratory chain complex deficiency with low activities of complexes I and IV. PCR-Sanger-sequencing confirmed the variant, which was estimated to have occurred 1550 years ago. These results establish the *MRPS23*-associated mitochondrial disorder inherited in an autosomal recessive pattern and provide insight into its clinical and metabolic features.

## Introduction

Mitochondrial disorders are caused by pathogenic variants in nuclear or mitochondrial genes leading to impaired ATP generation and inadequate energy production. They have a worldwide prevalence of 5 to 20 in 100,000^1^ and present with a variety of symptoms including growth failure, lactic acidosis, neuropathy, cardiomyopathy, hepatopathy, and myopathy^2^. Although variants in at least 343 genes have been reported to cause mitochondrial disorders (http://genomit.eu/), evidence for some genes as disease-causing is limited, and their clinical and metabolic features are not well-described.

In a previous study by Kodha et al. in 2016, a missense variant in the *MRPS23* gene was identified in a Japanese patient with hypoglycemia, liver dysfunction, and combined respiratory chain complex deficiency with low levels of complexes I and IV activities^3^. To date, this is the only report of an *MRPS23* germline variant, highlighting the need for more patients and detailed clinical and metabolic features to establish *MRPS23* as a disease gene and understand its phenotypic spectrum. In this study, we identified eight patients from five Hmong families with the same *MRPS23* c.119C>T; p.P40L germline variant and characterized their clinical manifestations and metabolic profiles from cultured fibroblasts.

## Materials and methods

### Patients and the obtainment of blood samples

Of 5134 exomes performed in our center, we identified five independent patients who were homozygous for the same germline variant c.119C>T; p.P40L in *MRPS23*. They were from five different medical centers in Thailand including Maharaj Nakhon Chiang Mai Hospital, Siriraj Hospital, Queen Sirikit National Institute of Child Health, Nakornping Hospital and King Chulalongkorn Memorial Hospital. Their medical records were reviewed. Their blood samples were obtained and sent to our center at Chulalongkorn University. All participants have been processed in accordance with the Declaration of Helsinki, and informed consents were obtained from all participants and their legal guardians. The study was approved by the Institutional Review Board of the Faculty of Medicine of Chulalongkorn University (IRB#264/62).

### Variant analysis

After performing whole exome sequencing on the DNA samples extracted from leukocytes, carried out by Macrogen Inc. (Seoul, Korea), and analyzing the data by filtering variants located in exons or flanking introns, as well as rare nonsynonymous variants with a minor allele frequency of less than 1% in the Genome Aggregation Database (gnomAD), Sanger sequencing was utilized to validate and analyze the identified variant, c.119C>T; p.P40L in *MRPS23* for all patients and their available family members.

### Variant dating

**Figure 1:**
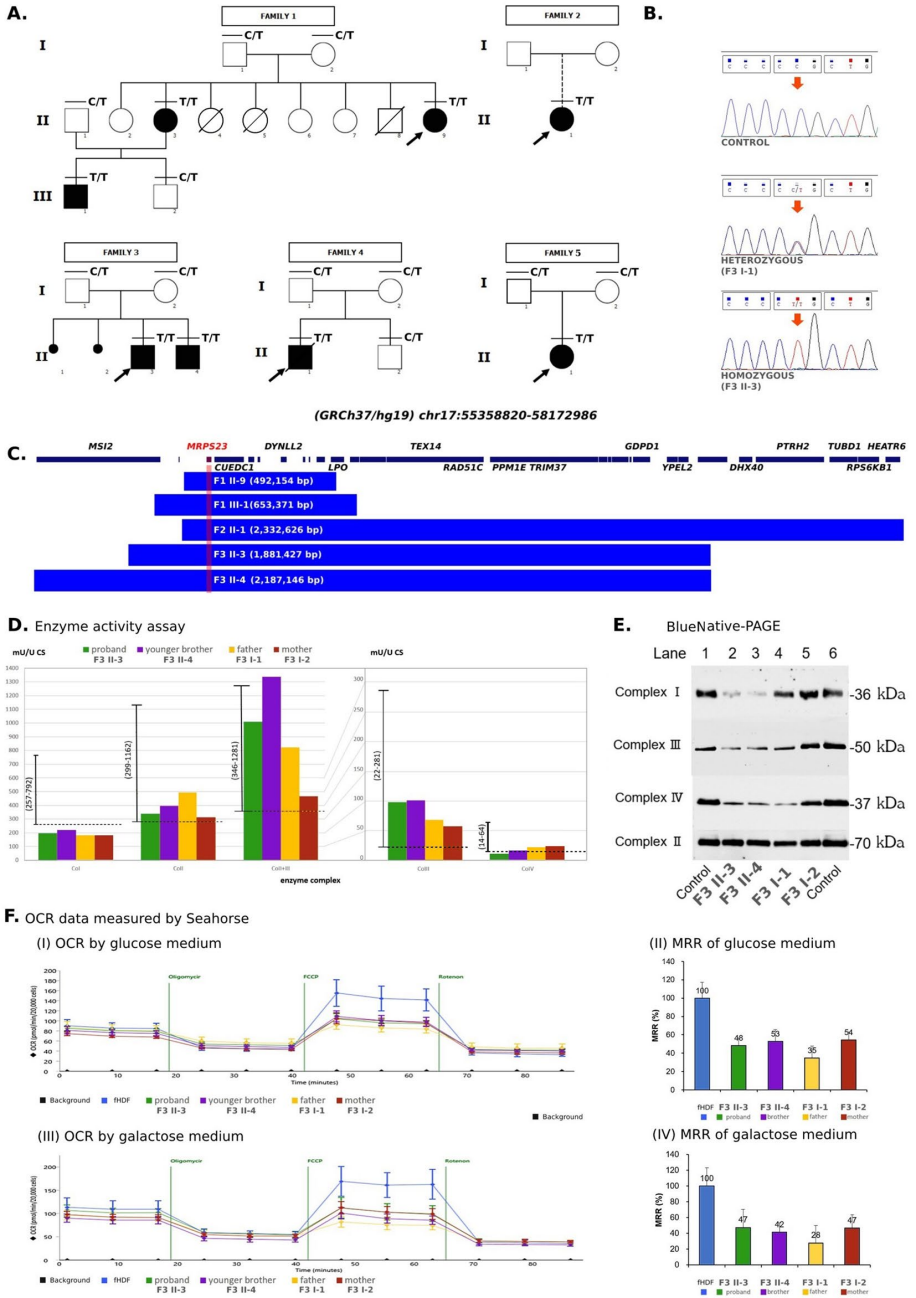
Genetic and metabolic features of the *MRPS23*-associated mitochondrial disorder. (**A**) Pedigrees of the five independent families. Dark boxes represented affected patients. (**B**) Chromatogram of the wild type, heterozygous and homozygous *MRPS23* c.119C>T; p.P40L variant. (**C**) Size of homozygous regions around the *MRPS23* c.119C>T; p.P40L variant in five patients (F1 II-9 and F1 III-1 in Family 1, F2 II-1 in Family 2, and F3 II-3 and F3 II-4 in Family 3). (**D**) Functional studies of fibroblasts from patients and their parents in Family 3. Mitochondrial respiratory chain enzyme activity. (**E**) The Blue Native-PAGE of protein lysate from mitochondrial’s fibroblasts. Twenty microgram of mitochondria from two controls and four patients were loaded in each lane; after electrophoresis by BlueNative-PAGE, they were transferred to PVDF membranes for western blotting. The membranes were cut into separate pieces prior to hybridization with antibodies for complex I (Invitrogen, MA, USA, 459100; NDUFA9 antibody), III (Invitrogen MA, USA, 459140; UQCRC1 antibody), IV (Invitrogen MA, USA, 459600; MTCO1 antibody), II (Invitrogen MA, USA, 459600; MTCO1 antibody) and were incubated with the respective antibodies for detection (see Supplementary Information 1). (**F**) Oxygen consumption rate (OCR) by Seahorse.

The SNP array using HumanOmniZhongHua-8 BeadChip was performed in 13 individuals (All individuals with a horizontal line above each individual symbol in Fig. 1A, except II-3 and III-2 in Family 1, II-2 in Family 4 and Family 5). The variant dating was estimated using a web tool https://shiny.wehi.edu.au/rafehi.h/mutation-dating/^4^.

### Skin fibroblast culture

The skin punch biopsy of the two affected siblings and their parents of Family 3 was performed. Skin fibroblasts were cultured at 37 °C and 5% CO_2_ in Dulbecco’s modified Eagle’s medium (DMEM with 4.5 g/L glucose; Nacalai Tesque; Kyoto, Japan) supplemented with 10% fetal bovine serum and 1% penicillin–streptomycin. Cells were harvested after they reached 80% confluence and used for enzyme activity assay.

### Enzyme activity assay

All these enzyme activity assay data were compared with citrate synthase, which is an intrinsic indicator of mitochondria and used to assess enzyme activity. Mitochondrial respiratory chain enzyme activity was analyzed using skin fibroblasts derived from patients and their parents of Family 3.

### The OCR (oxygen consumption rate) by Seahorse and BlueNative-PAGE from skin fibroblasts

The protocol from Ogawa et al. and Invernizzi et al. was used for cell preparation and OCR experiment^5,6^. The mitochondrial oxygen consumption of skin fibroblasts derived from *MRPS23* patients and fHDF (fetal human dermal fibroblast) as a control was measured, and the maximum respiratory rate (MRR) was compared. Blue Native-PAGE was performed to fractionate protein complexes in their native state. To enable probing for proteins of a specific molecular weight range, Western blot membranes were cut into separate pieces prior to antibody hybridization.

### Declaration

During the preparation of this work the authors used https://chat.openai.com in order to improve language and readability. After using this tool, the authors reviewed and edited the content as needed and take full responsibility for the content of the publication.

## Results

### Clinical characterization of patients with homozygous c.119C>T; p.P40L variant in *MRPS23*

The clinical characteristics of all eight individuals from five independent families were presented in Table 1. They all were of Hmong hill tribe. Since some lived in a very remote area with limited access to medical care, their clinical information was incomplete. One patient (F4 II-1) died at age 2 years and 4 months due to fever from an unknown cause, which might be also resulted from limited medical access. Seven patients homozygous for the variant manifested with alteration of consciousness and recurrent vomiting, except individual F3 II-4, who was diagnosed pre-symptomatically after his older brother (F3 II-3) was given a molecular diagnosis. Other clinical features included delayed growth (50%), delayed development (60%), hearing impairment (33%), hypoglycemia (67%), lactic acidosis (83%) and transaminitis (40%). Ebstein anomaly, a congenital heart defect, was found in one patient, F3 II-4. In addition, one proband, F4 II-1, underwent an MRI scan of the brain, which showed no appreciable abnormalities.

**Table 1:** The clinical characteristics of five probands and three other affected family members.

Clinical characteristics	F1 II-3	F1 II-9	F1 III-1	F2 II-1	F3 II-3	F3 II-4	F4 II-1	F5 II-1	Total 8 affected patients
Sex	Female	Female	Male	Female	Male	Male	Male	Female	F:M = 4:4
Age at last follow-up	17 years	4 years 6 months	1 year	7 years 6 months	4 y 3 months	1 year 4 months	2 years 4 months	3 years 2 months	1–17 years
Age of onset	1 year	6 months	6 months	2 years	1 year 11 months	1 year	11 months	10 months	6 months–1 year 11 months
Presenting symptoms	Alteration of consciousness with recurrent vomiting	Alteration of consciousness with recurrent vomiting	Alteration of consciousness with recurrent vomiting	Alteration of consciousness with recurrent vomiting	Alteration of consciousness and vomiting	Presymptomatic diagnosis	Alteration of consciousness and vomiting	Recurrent hypoglycemia with wide gap metabolic acidosis during fever/nausea, vomiting	Alteration of consciousness and vomiting
Status at last visit	Alive	Alive	Alive	Alive	Alive	Alive	Dead at 2 years 4 m due to fever from an unknown cause	Alive	Alive:dead = 7:1
Delayed growth	NA	N	NA	N	Y	Y	Y	N	3/6 (50%)
Delayed development	NA	N/A	NA	Y (mildly)	N	Y	Y	N	3/5 (60%)
Hearing impairment	NA	N/A	NA	Y (severely affected)	N	N	NA	NA	1/3 (33%)
Hypoglycemia (lowest level; mg%)	NA	Y (33)	NA	Y	Y (25)	N	N	Y (15)	4/6 (67%)
Serum HCO3 (mEq/L)	NA	7	NA	NA	7.4	NA	17	7	7–17
Anion gap	NA	24	NA	NA	21.6	NA	20	23	20–23
Lactic acidosis (mmol/L)	NA	Y (6.03)	NA	Y (4.3)	Y (7.5)	N	Y (6)	Y (8.1)	5/6 (83%)
Transaminitis (AST, ALT (U/L:N < 45))	NA	Y (52, 22)	NA	Y (332, 138)	N	N	N	NA	2/5 (40%)
Additional findings		Hyperammonemia (51.6 mcg/dL)				Ebstein anomaly			

### Variant studies

Whole exome sequencing revealed that all five probands were homozygous for the c.119C>T; p.P40L variant in *MRPS23*. Among the 10,268 alleles analyzed from our dataset of 5134 exomes, only a single heterozygous *MRPS23* c.119C>T allele was identified in the unaffected father of a patient with a chromosome abnormality hailing from Chiang Mai province. Based on the family name, there is a possibility that he belongs to the Hmong Clans. PCR-Sanger sequencing confirmed its presence and identified more patients. Genotype of each individual is shown in Fig. 1A and exemplified in Fig. 1B.

### Variant dating

Haplotype sharing of five patients was shown in Fig. 1C. The homozygous regions spanned around 500–2000 kb. The age of this haplotype following Gandolfo LC’s method was estimated to be 62.1 generations ago, with a confidence interval of 22–187.2. Assuming 25 years per generation, this variant occurred 1550 years ago.

### Enzyme activity assay, the OCR (oxygen consumption rate) by Seahorse and BlueNative-PAGE from skin fibroblasts

The enzyme activity assay of OXPHOS enzyme complexes I, II, II + III, III and IV was presented in Fig. 1D. Mitochondrial respiratory chain enzyme activity was analyzed using skin fibroblasts derived from two patients and their parents in Family 3. The enzyme activity measurements were confirmed based on the diagnostic criteria described in the literature^7,8^. The range of 40–100% (black line with upper and lower range in Fig. 1D) was considered as the reference range, and the activity was considered to be decreased if it was below 40%. As shown in Fig. 1D, the enzymatic activity of complex I in fibroblasts of the two patients and their parents and that of complex IV in II-3 were found to be decreased compared to the reference ranges.

For BlueNative-PAGE, mitochondria were isolated from fibroblasts of patients and their parents in Family 3. As the result in Fig. 1E, complexes I, III, and IV band concentrations of both patients were decreased compared to the normal control. In their mother, only the complex IV band concentration was decreased. In their father, there was a slight decrease in the concentration of complex I band, but a decrease in the concentration of complexes III and IV bands. However, no decrease in the concentration of complex II band was observed.

The mitochondrial oxygen consumption of skin fibroblasts derived from the two patients and their parents in Family 3 and fHDF as a control was measured. The bar graph (Fig. 1F) was a comparison of the maximum respiratory rate (MRR) of fibroblasts, and it was found that the OCR of *MRPS23*-derived fibroblasts was considerably lower than that of control cells in both glucose and galactose medium.

## Discussion

In this article, we provide a comprehensive description of the clinical, metabolic, and genetic features of the *MRPS23*-related mitochondrial disorder. While a previous study identified a patient with the c.119C>G; p.P40R variant in the *MRPS23* gene, there was limited information on phenotypic features. We present eight additional individuals homozygous for a pathogenic variant, confirming *MRPS23* as a gene causing mitochondrial disorder, and provide a more detailed account of the phenotypic spectrum and metabolic disturbances.

The *MRPS23* gene, located on chromosome 17q22, encodes a protein component of the mitoribosome small subunit, which plays a role in mitochondrial translation^9^. Deficiencies in complexes I, II, III, and IV are the most common causes of mitochondrial OXPHOS deficiency. Moreover, mitoribosome deficiency can also cause mitochondrial disease^10^. Variants in other members of the MRPS family, such as bi-allelic *MRPS34* and *MRPS2* variants, can cause Leigh-like syndrome and OXPHOS deficiency^3,11^.

Our study identified eight individuals from five independent families who were homozygous for the c.119C>T; p.P40L variant in *MRPS23*. Their available parents were found to carry one mutant allele, indicating an autosomal recessive mode of inheritance. All five families had Hmong ancestry, and the fact that they share the same variant suggests a founder effect. Despite clan exogamy being the primary kinship principle, ethnic endogamy in the Hmong population resulted from cultural preference could lead to the accumulation of variants of autosomal recessive disorders^12,13^. Using long homozygous regions, we estimated that the variant occurred 62.1 generations or 1550 years ago.

The clinical features of these eight patients are typical of disorders caused by variants in genes encoding mitoribosomal subunits^10^. These include delayed growth, delayed development, hearing impairment, hypoglycemia, lactic acidosis, and transaminitis. Notably, not all patients had delayed growth or development, indicating that with proper management, patients with *MRPS23*-related disorder could have normal growth and development. Although presymptomatic diagnosis in individual F3 II-4 was expected to yield an even more favorable outcome, growth and developmental delay were observed due to the co-occurrence of congenital heart disease.

There are several lines of evidence to demonstrate that the variant in the nuclear *MRPS23* gene is responsible for the disease. First, all probands with similar clinical findings were homozygous for the same *MRPS23* c.119C>T variant, while there were no other individuals in our in-house exome database who were homozygous of the variants. Secondly, the *MRPS23* variant segregates with the disease in all identified five families. All parents were heterozygous and none of the unaffected members were homozygous for the variants. Thirdly, the amino acid residue 40 is likely to impact protein functions, as supported by a previously reported patient with a pathogenic p.P40R variant at the same codon. Fourthly, this variant was predicted to be pathogenic by six different prediction software tools. Specifically, it received a “deleterious” designation with a score of 0.00 from SIFT, a “probably damaging” classification with a score of 1.000 from PolyPhen-2, a “possibly pathogenic” assessment with a score of 0.038 from MCAP, and was considered “most likely to be deleterious and potentially pathogenic” with a score of 26.8 (exceeding the threshold of 20) according to CADD. Additionally, it was labeled as “pathogenic strong” with a score of 0.875 by MutPred and deemed “disease causing” by MutationTaster2.

This variant is also classified as likely pathogenic (PP3 PM5 PM2 PP5) according to the American College of Medical Genetics and Genomics (ACMG) classification system. Fifthly, the proline at amino acid residue 40 is highly conserved from c. elegans to humans, as shown by evolutionary conservation analysis performed by the MutationTaster2 software^14^ (see Supplementary Information 2).

To determine metabolic disturbances, we used skin fibroblasts from patients (homozygous TT) and parents (heterozygous CT) in Family 3 for enzyme activity assays, oxygen consumption rate by Seahorse, and BlueNative-PAGE experiments. No induced-mutation cells were used for functional studies in this report. Western blotting experiments showed decreased band intensities of complex I in both patients and those of complex IV in both patients and their father compared to the control. Complex II did not show any difference among patients, their parents and controls. This is probably because complex II, unlike complexes I, III, IV, and V, does not contain any subunits which are encoded by mitochondrial genome^3,10^. Both patients and their parents exhibited similar aberrations in the enzyme activity assay, BlueNative-PAGE, OCR by glucose medium, and MRR of glucose medium; therefore all these assays cannot differentiate patients carrying biallelic loss-of-function variants from carriers who carry monoallelic variants. Only the Western blot determining the complex I level can distinguish patients from carriers. This situation resembles the acid beta-glucosidase enzyme activity test used in Gaucher disease^15^. Since the complex I deficiency was detected in the parents, determination of the complex I cannot be used to diagnosed *MRPS23*-associated mitochondrial disorder. This emphasizes the importance of molecular diagnosis.

This study highlights the specific *MRPS23* variant associated with lactic acidosis and hypoglycemia, which has significant implications for individuals of Hmong ancestry carrying the homozygous c.119C>T; p.P40L variant. Symptomatic considerations and pre-symptomatic warnings are both vital in clinical management. Given the high prevalence of this variant, it is crucial to prioritize *MRPS23*-related disorder as the primary diagnosis when Hmong patients present symptoms of lactic acidosis and hypoglycemia.

In conclusion, we establish *MRPS23* as a nuclear gene causing a mitochondrial disorder inherited in an autosomal recessive manner and characterize its clinical manifestations and metabolic profiles.

### Supplementary Information

Supplementary Information 1.

Supplementary Information 2.

## Data Availability

This variant was submitted into the ClinVar database (Accession SCV004015155). The datasets generated during and analyzed during the current study are available from the corresponding author on reasonable request.
