# Supplementary material for: Genetic, metabolic and clinical delineation of an MRPS23-associated mitochondrial disorder
Source: Sci Rep. 2023 Dec 12;13:22005. doi: 10.1038/s41598-023-49161-7 (PMC10716371; doi:10.1038/s41598-023-49161-7)

**Supplementary Information 1**

Regarding the processed and cropped images of the BlueNative-PAGE result in Figure 1E, original images of full-length blots were not provided because the Western blot membranes were cut into separate pieces prior to hybridization with antibodies to allow each piece to be probed for proteins of a specific molecular weight range. However, original images of all blots for complexes I, III, IV, and II, in the order presented in Figure 1E, with membrane edges visible, are included below: **a.** Complex I; **b-1.** Complex III on the same blotting membrane as presented in Figure 1E, with less exposure time and **b-2.** increased exposure time for chemiluminescent Western blot detection; **c.** Complexes IV and II (shown on the same blotting membrane, with Complex IV in the upper bands and Complex II in the lower bands. These blots were later cropped and presented separately in Figure 1E.) Note that the rightmost lane in each photo, marked with an asterisk (*, Lane 7), was tested with half the amount of protein as a comparison but was excluded from the final results shown in Figure 1E. F3 II-3, F3 II-4, F3 I-1, and F3 I-2 represent the affected proband, affected brother, father, and mother in family 3 (F3), respectively.

**a.**


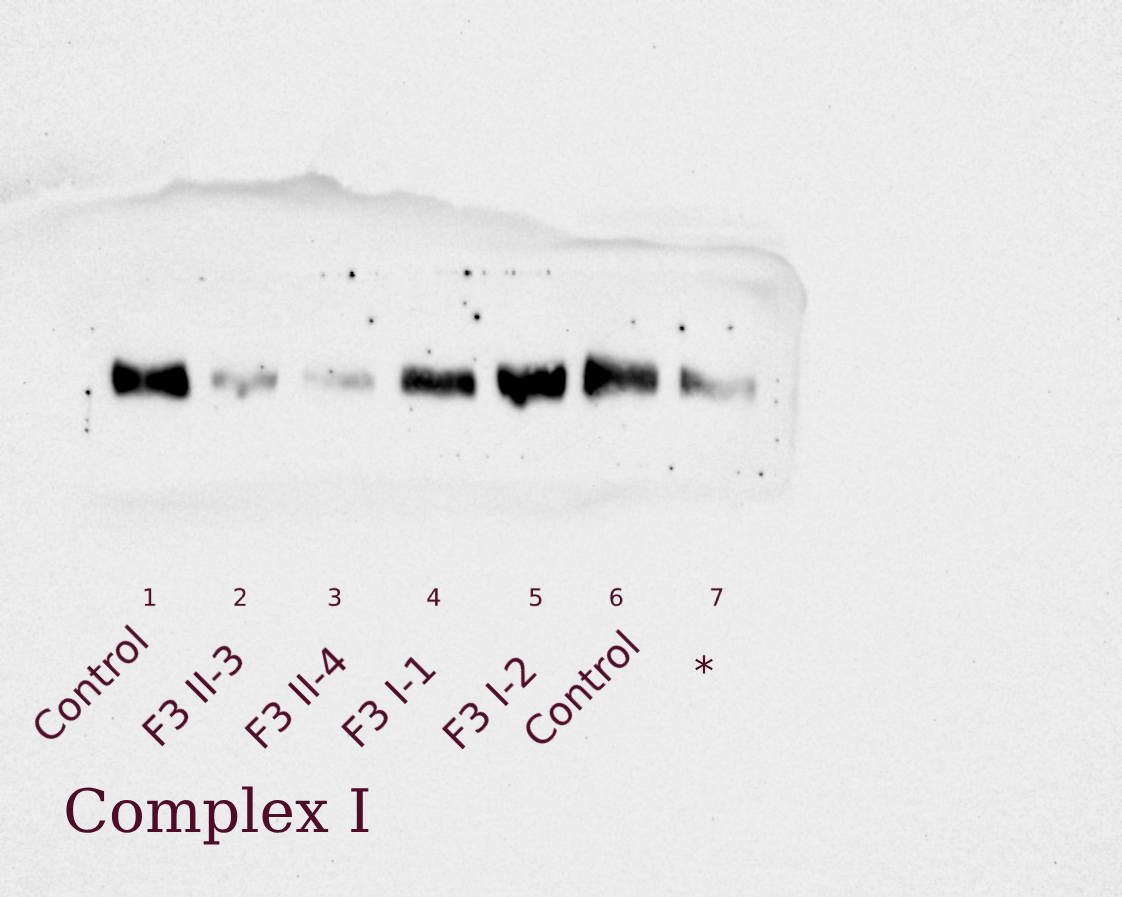


**b-1.
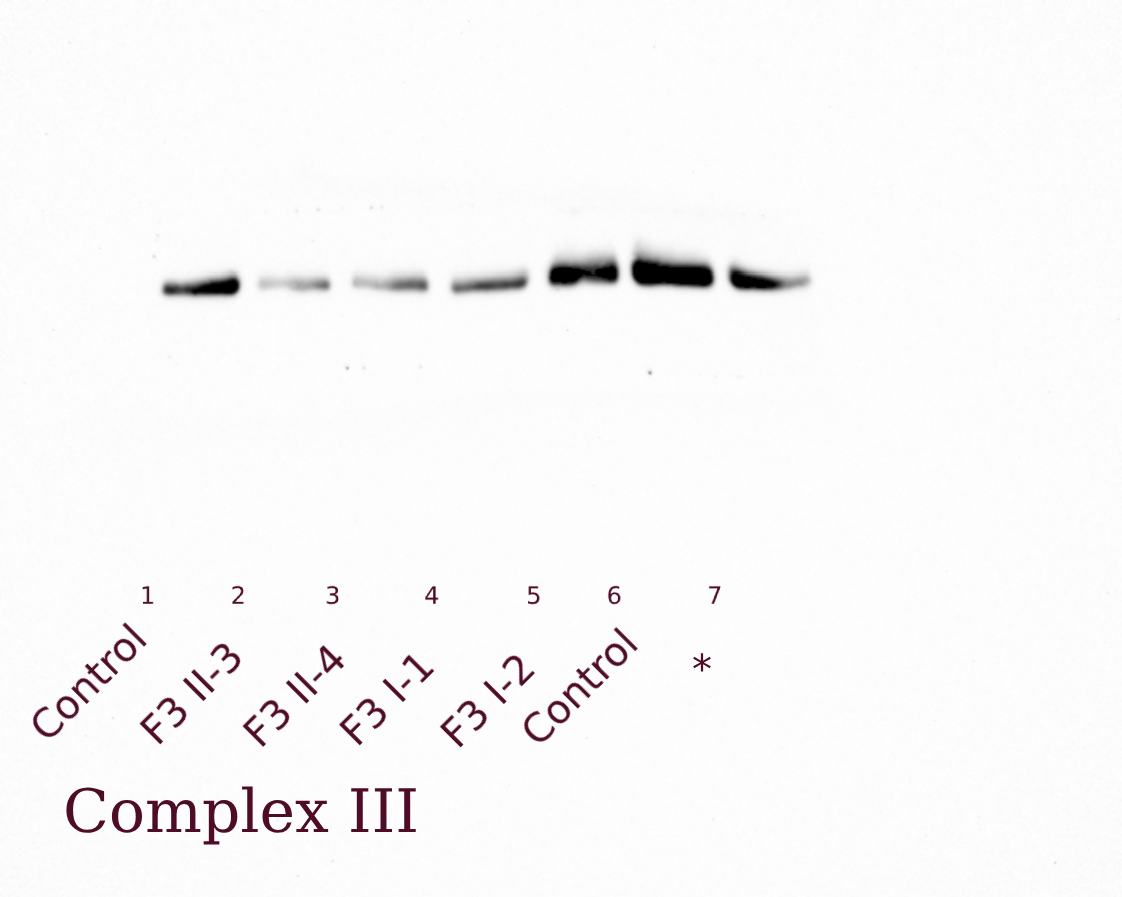
**

**b-2.**


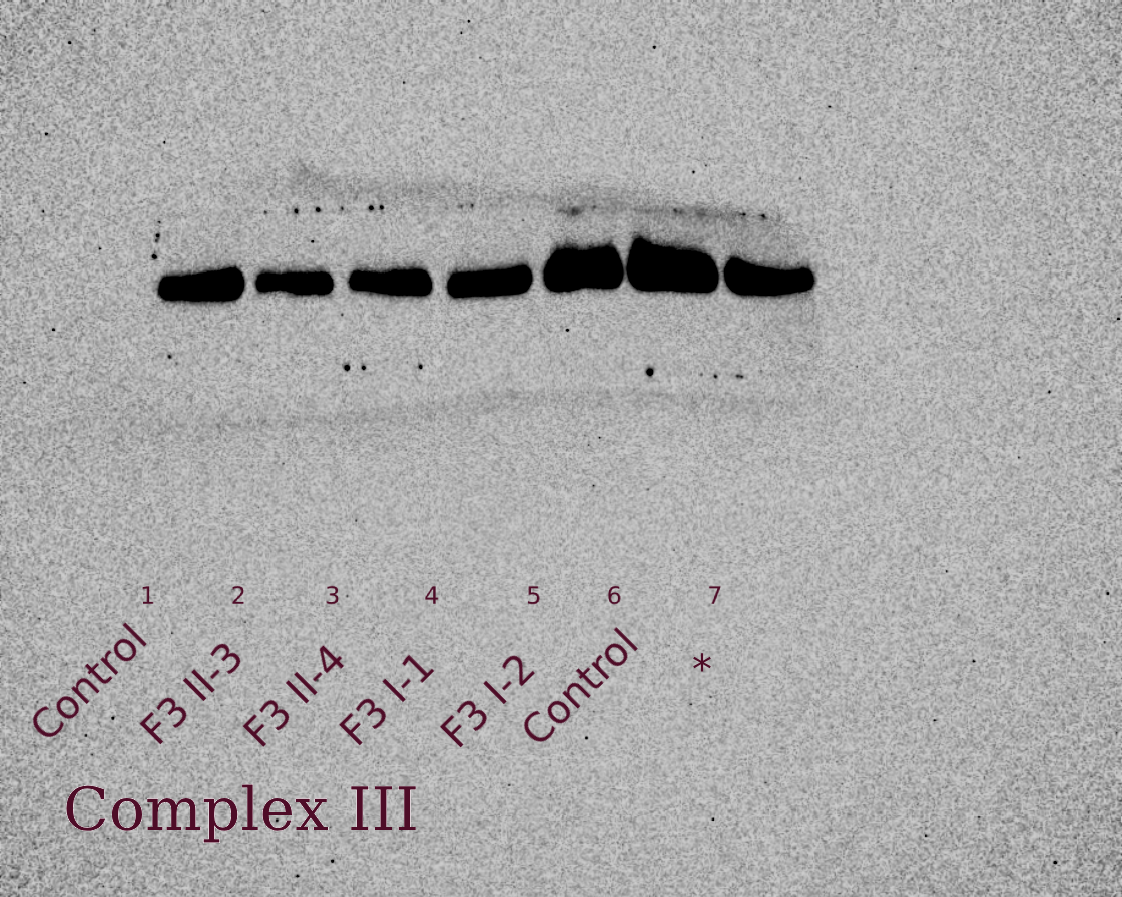


**c.**


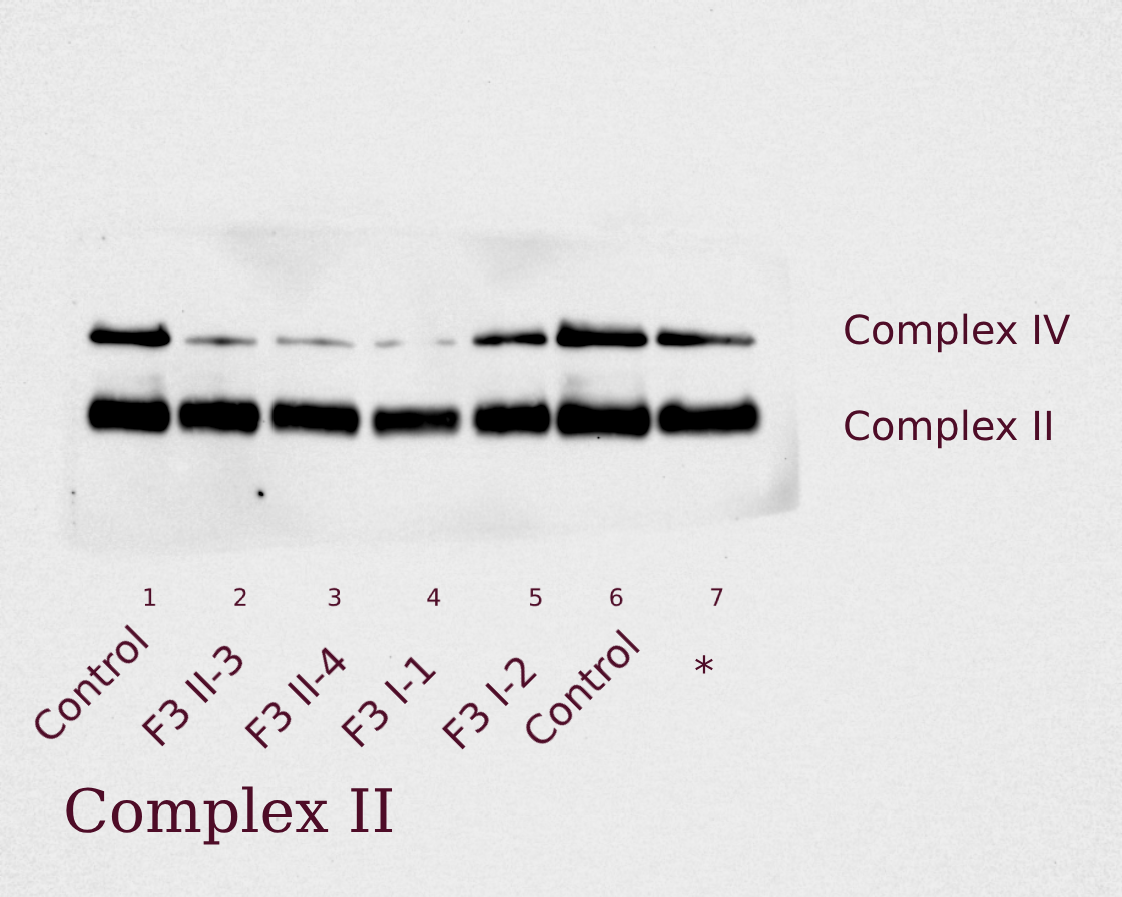

Supplement: Supplementary file 1 — Supplementary Information 1. [file 41598_2023_49161_MOESM1_ESM.docx]
