# Supplementary material for: Genetic, metabolic and clinical delineation of an MRPS23-associated mitochondrial disorder
Source: Sci Rep. 2023 Dec 12;13:22005. doi: 10.1038/s41598-023-49161-7 (PMC10716371; doi:10.1038/s41598-023-49161-7)

**Supplementary Information 2**

Conservation analysis of the *MRPS23*: c.119C>T; p.P40L variant reveals a high degree of conservation for the proline-40 residue, from *C. elegans* to humans. This analysis was conducted using the MutationTaster2 software ^14^.


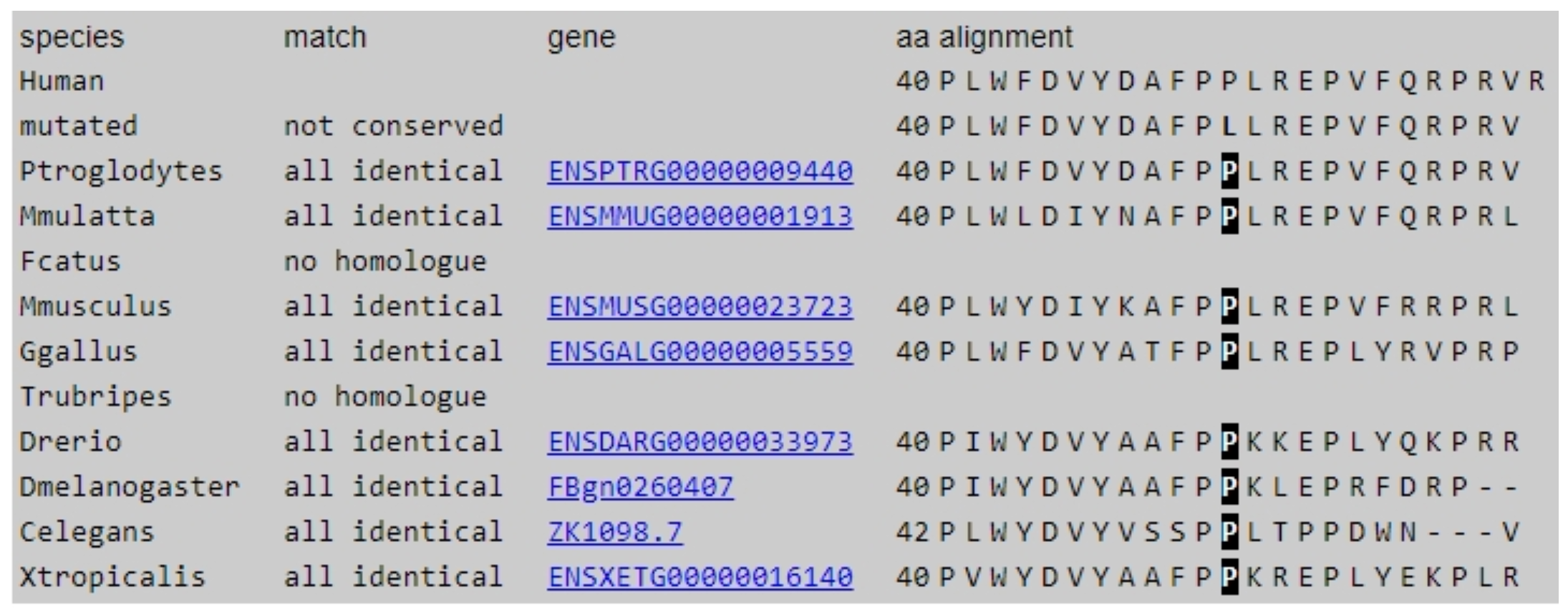

Supplement: Supplementary file 2 — Supplementary Information 2. [file 41598_2023_49161_MOESM2_ESM.docx]
